# Use of mobile technology by frontline health workers to promote reproductive, maternal, newborn and child health and nutrition: a cluster randomized controlled Trial in Bihar, India

**DOI:** 10.7189/jogh.09.020424

**Published:** 2019-12

**Authors:** Suzan L Carmichael, Kala Mehta, Sridhar Srikantiah, Tanmay Mahapatra, Indrajit Chaudhuri, Ramkrishnan Balakrishnan, Sharad Chaturvedi, Hina Raheel, Evan Borkum, Shamik Trehan, Yingjie Weng, Rajani Kaimal, Anitha Sivasankaran, Swetha Sridharan, Dana Rotz, Usha Kiran Tarigopula, Debarshi Bhattacharya, Yamini Atmavilas, Kevin T Pepper, Anu Rangarajan, Gary L Darmstadt, the Ananya Study Group

**Affiliations:** 1 Department of Pediatrics, Stanford University School of Medicine, Stanford, California, USA; 2 Center for Population Health Sciences, Stanford University School of Medicine, Stanford, California, USA; 3 CARE India, Patna, India; 4 Current affiliation: Project Concern International, Delhi, India; 5 Mathematica Policy Research, Princeton, New Jersey, USA; 6 Current address: Dr. Reddy’s Foundation, Hyderabad, India; 7 Quantitative Sciences Unit, Department of Medicine, Stanford University, Stanford, California, USA; 8 India Country Office, Bill and Melinda Gates Foundation, Delhi, India; * Membership of the Ananya Study Group is provided in the Acknowledgements

## Abstract

**Background:**

mHealth technology holds promise for improving the effectiveness of frontline health workers (FLWs), who provide most health-related primary care services, especially reproductive, maternal, newborn, child health and nutrition services (RMNCHN), in low-resource – especially hard-to-reach – settings. Data are lacking, however, from rigorous evaluations of mHealth interventions on delivery of health services or on health-related behaviors and outcomes.

**Methods:**

The Information Communication Technology-Continuum of Care Service (ICT-CCS) tool was designed for use by community-based FLWs to increase the coverage, quality and coordination of services they provide in Bihar, India. It consisted of numerous mobile phone-based job aids aimed to improve key RMNCHN-related behaviors and outcomes. ICT-CCS was implemented in Saharsa district, with cluster randomization at the health sub-center level. In total, evaluation surveys were conducted with approximately 1100 FLWs and 3000 beneficiaries who had delivered an infant in the previous year in the catchment areas of intervention and control health sub-centers, about half before implementation (mid-2012) and half two years afterward (mid-2014). Analyses included bivariate and difference-in-difference analyses across study groups.

**Results:**

The ICT-CCS intervention was associated with more frequent coordination of AWWs with ASHAs on home visits and greater job confidence among ASHAs. The intervention resulted in an 11 percentage point increase in FLW antenatal home visits during the third trimester (*P* = 0.04). In the post-implementation period, postnatal home visits during the first week were increased in the intervention (72%) vs the control (60%) group (*P* < 0.01). The intervention also resulted in 13, 12, and 21 percentage point increases in skin-to-skin care (*P* < 0.01), breastfeeding immediately after delivery (*P* < 0.01), and age-appropriate complementary feeding (*P* < 0.01). FLW supervision and other RMNCHN behaviors were not significantly impacted.

**Conclusions:**

Important improvements in FLW home visits and RMNCHN behaviors were achieved. The ICT-CCS tool shows promise for facilitating FLW effectiveness in improving RMNCHN behaviors.

Use of mobile technology in low and middle-income countries (LMICs) has increased rapidly in recent years. Mobile phone subscriptions are now nearly as numerous as people in the world (7.7 billion worldwide) [1], and this expanded access has the potential to positively impact health care delivery in LMICs [2,3]. Commonly referred to as mHealth, these applications involve the use of mobile communication devices such as mobile phones, personal digital assistants and tablet computers to facilitate and record data on the provision of information and health services and on population health status. mHealth is particularly promising for improving the effectiveness of frontline health workers (FLWs), who provide most primary health care services, especially in hard-to-reach, low-resource settings. As demonstrated by prior studies, mHealth tools may enhance the effectiveness of FLWs by improving tracking and service provision, the completeness and equity of coverage of their target populations, and the quality and consistency of the health-related information they provide [3-5].

FLWs contribute to the continuum of care by addressing a variety of issues such as family planning, maternal health, newborn care, infant feeding, and immunization. Successful mHealth interventions have included improvements in antenatal care attendance in Rwanda and Nigeria [6,7], infant growth monitoring in Kenya [8], and breastfeeding in Malawi and China [9,10]. Most prior studies were limited to testing feasibility and acceptability, but they do suggest that mHealth tools have the potential to improve health care delivery and data collection, with adequate attention to training and implementation [11,12]. More research is needed that rigorously evaluates mHealth impacts on health behaviors and outcomes [5,13], and that demonstrates effective, efficacious, scalable and sustainable ways to use mHealth to support the FLW's role in information and service delivery.

This paper evaluates the impact of a novel mHealth tool that was implemented in Bihar, one of India’s poorest and most populous states (104.1 million, 88.7% rural) which relies heavily on FLWs to provide community-level reproductive, maternal, newborn and child health and nutrition (RMNCHN)-related services [14]. The tool – the Information Communication Technology Continuum of Care Service (ICT-CCS) – was designed for use by FLWs to increase the coverage, quality and coordination of the services that they provide; enhance and align their communications with beneficiaries; and facilitate their supervision, with the ultimate goal of improving RMNCHN-related outcomes. It was implemented in the catchment areas of 70 health sub-centers in the Saharsa district of Bihar, with cluster randomization at the health sub-center level. We examined primary outcomes reflecting maternal RMNCHN-related behaviors and care, pertinent to the antenatal, delivery and newborn periods, infant feeding, immunizations, and family planning. To further our understanding of the potential impact of the tool on FLWs’ performance, we examined as secondary outcomes FLWs’ use of the tool, coordination amongst each other, and supervision.

## METHODS

### Study context – The *Ananya* program

The ICT-CCS intervention was introduced as a supplement to reinforce the delivery of the health messages of the *Ananya* program, which was concurrently implemented throughout Saharsa and seven other districts in Bihar. *Ananya*’s long-term goal was to reduce rates of maternal, newborn, and child mortality, fertility, and child undernutrition statewide in Bihar by improving behaviors related to family planning, antenatal care (ANC) and delivery preparation, postnatal care, complementary feeding, and child immunization. *Ananya* included an integrated set of multi-level interventions to reach these goals. Thus, the estimated impacts of the ICT-CCS intervention are interpreted as the added value of this mHealth tool beyond that of core *Ananya* program interventions. The basic content being administered via the ICT-CCS tool was no different than *Ananya*; the purpose of this intervention was to determine whether the ICT-CCS mobile health tool could enhance FLW performance and health impact by facilitating access to relevant content, beneficiary tracking, delivery of appropriate services, and supervision at scale in a low-resource, remote setting.

### Study design – ICT-CCS intervention

**Figure 1:**
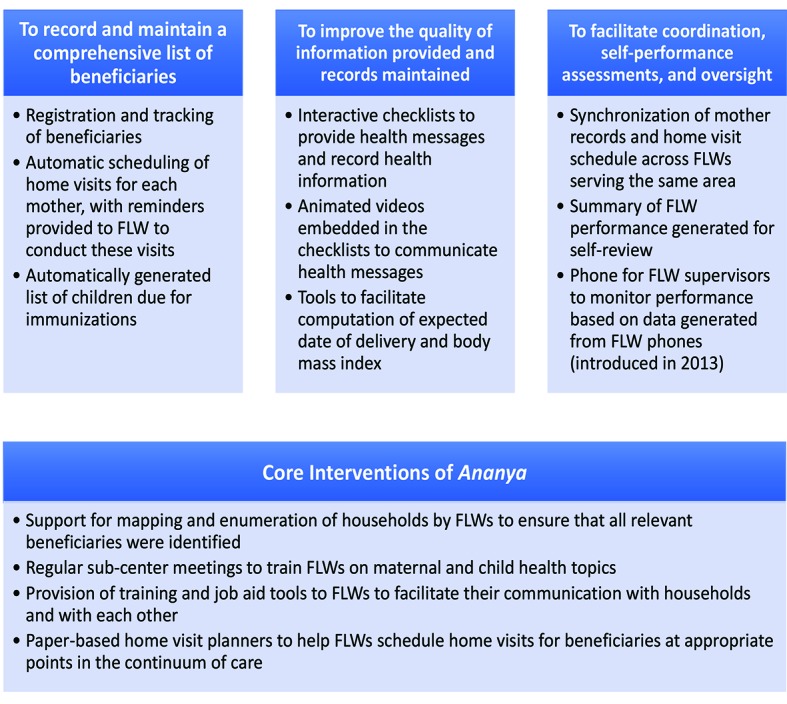
Description of core features of the ICT-CCS intervention by objective, and the core interventions of the *Ananya* program that were supported by the ICT-CCS intervention. ICT-CCS – Information Communication Technology Continuum of Care Service, FLW – frontline worker.

**Figure 2:**
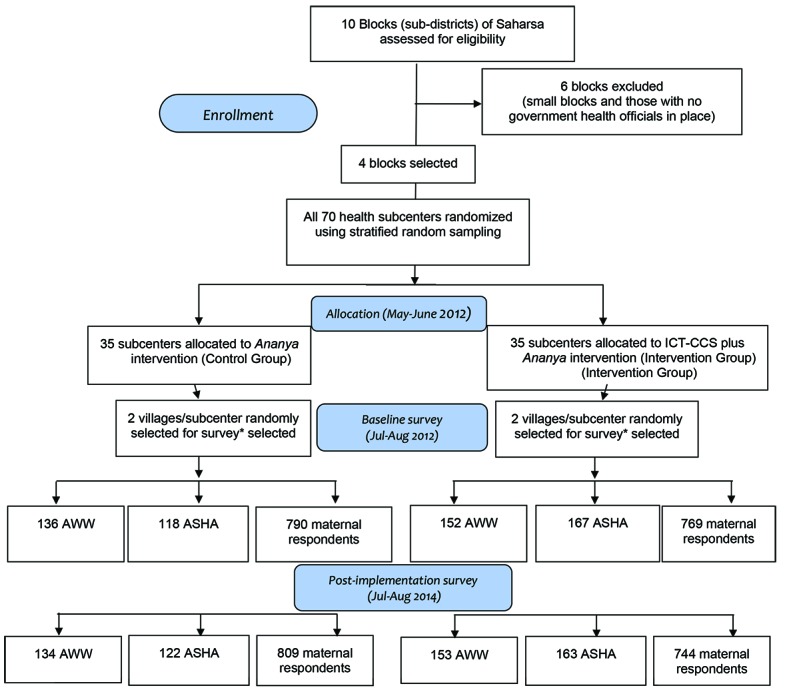
Description of the design and sample selection for the ICT-CCS (Information Communication Technology Continuum of Care Service) intervention trial in Saharsa, Bihar 2012-2014. *If a large village (≥150 households, as identified by CARE) was selected, we then organized the village into approximately equal-sized segments (75 to 150 households per segment) and randomly selected one segment of the village for surveying.

The ICT-CCS tool, which was administered via a feature phone with a keyboard provided to each FLW, integrated a uniquely comprehensive set of functions to assist FLWs in their duties, including registration and tracking of beneficiaries, automated scheduling of home visits, provision of health information through videos, guided protocols for conducting home visits through checklists, a feature to track child immunizations, and supervisory tools. The tool was designed to enhance FLW-maternal interactions during the “thousand days” of pregnancy through a child’s second birthday, addressing the continuum of family health care encompassed by RMNCHN. **Figure 1** provides further detail regarding the ICT-CCS tool and key interventions of the *Ananya* program, and **Figure 2** provides an overview of the intervention delivery and evaluation design. The ICT-CCS intervention was administered by CARE India. In addition to CARE, multiple other organizations took important roles in the implementation of different components of the *Ananya* interventions.

The unit of random assignment for the ICT-CCS intervention was the health sub-center – the lowest-level public health facility – that covers several villages and their FLWs (a population of approximately 8000-12 000 per subcenter). Sample size calculations conducted during the study design phase suggested that a survey sample of 70 sub-centers and 20 women and 8 FLWs per sub-center would enable detection of changes of 6 to 10 percentage points in key maternal behaviors targeted by the intervention (assuming 80% power and α = 0.05) [15]. To limit implementation costs and logistical requirements, the intervention focused on four out of ten blocks (or sub-districts) in Saharsa. All 70 health sub-centers in these blocks were randomly assigned into equal-sized intervention and control groups (35 sub-centers each), using a stratified random assignment procedure based on the number of village-level health centers (known as Anganwadi Centers) served by the sub-center, which served as a proxy for the size of the population served. The stratification helped ensure that the intervention and control groups were balanced in the size of the population served, and reduced variance (and hence increased statistical power) in the analysis.

The primary participants in this intervention were two types of FLWs: the Accredited Social Health Activist (ASHA) and the Anganwadi Worker (AWW). ASHAs (from the Ministry of Health and Family Welfare, MoHFW) and AWWs (from the Ministry of Women and Child Development, MWCD) share a broad mandate for supporting a range of RMNCHN services, in addition to some services that are specific to one or the other FLW/department. The ASHA supports antenatal, childbirth and newborn care, and child immunizations as part of MoHFW services. The AWW focuses on preschool education and supplemental nutrition for pregnant women and young children through the Integrated Child Development Services (ICDS) scheme. Each village (or village segment, in large villages) is typically served by one ASHA and one AWW, who live in the community. For the purposes of *Ananya*, they had the same supervisor (either a Lady Supervisor or an Auxiliary Nurse Midwife, ANM) as efforts were made to facilitate planning and actions as a team. An important objective of the ICT-CCS tool was to facilitate interaction between ASHAs and AWWs, in hopes that this interaction would improve their effectiveness. CARE provided extensive training in the use of the ICT-CCS tool at health sub-centers, which consisted of 16 sessions of approximately 3 hours each over a period of 8 weeks. A substantial part of this training was aimed at enabling the FLWs to become comfortable in the use of the algorithm-loaded mobile phones (Nokia C2-01), including using the keyboard to type text such as the names of mothers and children and simple responses to questions such as Y or N.

The intervention was introduced in mid-2012, but the various components of the tool were rolled out in stages so that the full set of applications was available to FLWs in late 2012 to early 2013. Mobile telephones for FLW supervisors to monitor FLW performance based on data generated from FLW phones were implemented last, from February to April of 2013. The complete date range for participant recruitment and follow-up was 1 January 2012 to 31 August 2014.

### Study design – ICT-CCS intervention evaluation

The evaluation was designed and implemented by Mathematica Policy Research, who conducted extensive pilot testing before launching the survey and worked with Sambodhi and Population Health Foundation of India to oversee the survey data collection and administration.

We estimated the impacts of the intervention using survey data collected from FLWs and a representative sample of women who had given birth in the catchment areas of the intervention and control sub-centers in the previous year. Surveys were conducted at baseline (May to June, 2012, before the intervention began) and post-implementation (July to August, 2014, approximately two years after implementation began). FLWs were asked about their use of the technology, participation in sub-center meetings, home visits, and services they provided. Mothers were asked about their interactions with the FLWs and health behaviors and practices related to the project’s goals, such as family planning, ANC and delivery preparation, postnatal care, child immunizations, and complementary feeding. Health-related behaviors reported by maternal respondents were considered the primary endpoints of the intervention.

Two villages per health sub-center were randomly selected for survey. In large villages, one segment of the village was then randomly selected, defining segments such that there were about 20 eligible household respondents per segment. Interviews were attempted with all eligible women. Because a different cohort of women gave birth in the 12 months before each survey, the baseline and post-implementation samples were predominately different women (ie, repeated cross-sections), but they were located in the same villages.

For the FLW surveys, at baseline the ASHAs and AWWs who served beneficiaries in the villages (or village segments) selected for the household surveys were interviewed. For the post-implementation survey, an attempt was made to interview all ASHAs and AWWs identified at baseline, plus any new ones serving households in the sampled communities. **Figure 2** describes the number of FLWs and mothers who were interviewed for the baseline and post-implementation surveys.

Response rates were generally high and similar for intervention and control groups and by time period. For the post-implementation surveys, 99% of households responded to the listing survey, and 90% of eligible women responded to the evaluation survey. Among FLWs, the response rates were 97% for AWWs and 92% for ASHAs.

### Analysis

We compared demographic characteristics of the ASHAs, AWWs and maternal respondents at baseline and post-implementation according to their intervention allocation, using appropriate bivariate tests (χ^2^ tests for categorical variables and *t* tests for continuous variables unless otherwise noted in the tables). FLWs from intervention villages were asked a variety of questions about training and usage of the phones, at post-implementation; we present percentages of FLWs with positive responses to these questions.

We conducted a series of regression models to examine information from FLWs and mothers on indicators related to the intervention. We generated survey-weighted percentages and counts, to account for the survey design. Logistic regression models were conducted for binary outcomes and linear regressions for count variables.

For comparisons involving information from FLWs, which was only collected post-implementation, we conducted regression models that accounted for village as the primary sampling unit and sub-center as the primary stratum within the sampling unit. Separate regression models were conducted for each outcome, for each cadre (AWW, ASHA); the independent variable for these models was treatment allocation (ICT-CCS + *Ananya* vs *Ananya* alone). Next, in order to estimate whether either cadre performed better with the intervention, we conducted a separate regression model for each outcome that included all FLWs and contained a term representing cadre (AWW or ASHA), a term representing treatment (intervention or control), and an interaction of these two terms; the *P* value for the interaction term reflects whether the difference in the outcome among control and intervention FLWs was significantly different by cadre (*P*-value <0.10 was considered statistically significant).

For comparisons involving information from maternal respondents, regression models were performed that accounted for village as the primary sampling unit and sub-center as the primary stratum within the sampling unit, and with proportional sampling weights at the maternal respondent/household level. Table S1 in **Online Supplementary Document** shows the number of maternal respondents from whom data was available for various outcomes. Separate regression models were conducted for each outcome, for each time point (baseline and post-implementation, if available); the independent variable for these models was treatment allocation, In order to estimate the effect of the intervention on a particular outcome, we conducted a separate regression model for each outcome that included all maternal respondents. These models each included time (baseline or post-implementation), treatment (intervention or control), and an interaction of these two terms, which is represented by the difference in difference (DID) estimator and captures the change in outcome during the intervention period that was attributable to the ICT-CCS intervention. The DID was considered significant if *P* < 0.10 because it is an interaction term [14]. These models accounted for potential confounding by maternal age, household size, whether a woman belonged to a scheduled caste or tribe, literacy, lack of formal education, having a Below Poverty Line (BPL) card (which qualified women for certain government benefits), and socioeconomic status (SES) quartile. SES quartiles were determined using coefficients and cutoffs from a principal components analysis that used the *Ananya* statewide 2012 baseline data (following the methodology of the National Family Health Survey’s wealth index) [16]. Quartiles are therefore relative to the 2012 statewide SES distribution for women who gave birth in the previous 12 months. The adjustment variables were chosen based on the presence of significant differences between intervention and control women at baseline or post-implementation.

*P*-values less than 0.05 were considered significant for non-interaction comparisons. Stata version 14 and SAS version 9.1 were used for all analyses, which were replicated independently in both software packages. Further information is included in a detailed Statistical Appendix which is included in **Online Supplementary Document**.

### Ethics

The trial was registered at clinicaltrials.gov (NCT03406221). The authors confirm that all ongoing and related trials for this intervention are registered. Ethical clearance for the overall *Ananya* intervention and evaluation, which included this study, was obtained from the Institutional Review Board of the Public Health Foundation of India, New Delhi, and from the Health Ministry’s Screening Committee (approved on August 18, 2011); partners involved in the original trial design are unable to access the original trial protocol. Verbal informed consent was obtained from all respondents. Data analysis at Stanford University was deemed exempt from oversight by the Stanford Institutional Review Board.

## RESULTS

### Demographic characteristics

Almost all ASHAs and AWWs lived in the village they served and were on average in their mid-30s (**Table 1**). Nearly all (≥95%) were Hindu, and among those who were Hindu, about two-thirds were in Other Backward castes; a slightly higher proportion of ASHAs were in a Scheduled Caste than AWWs. The median standard of education was 12th grade for AWWs and 10th grade for ASHAs. Demographic characteristics for ASHAs and AWWs were well balanced between the control and intervention FLWs for the 2012 and 2014 evaluations.

**Table 1:** Demographic characteristics of Anganwadi Workers (AWW), Accredited Social Health Activists (ASHA), and maternal household respondents as part of the ICT-CCS intervention trial in Saharsa, Bihar, 2012-2014*

	Baseline (May-June 2012)			Post-implementation (July-August 2014)		
Control	Inter-vention	*P*-value	Control	Inter-vention	*P*-value
**AWWs**	n = 136	n = 152		n = 134	n = 153	
Lives in village she serves (%)	97	97	1.00	98	97	1.00
Mean age in years (SD)	35.5 (6.8)	35.5 (6.1)	0.49	36.0 (6.2)	36.6 (6.5)	0.48
Hindu (%)	97	97	1.00	97	97	0.85
Caste (among Hindus only) (%):						
-Scheduled caste/tribe (SC/ST) (lowest caste)	9	3	0.09	8	3	0.22
-Other backward caste (socially and educationally disadvantaged)	62	72		68	69	
-General caste	29	25		24	28	
Highest grade education standard (median, IQR) †	12 (2)	12 (2)	0.72	12 (4)	12 (0)	0.88
**ASHAs**	n = 118	n = 167		n = 122	n = 163	
Lives in village she serves (%)	98	98	1.00	99	99	1.00
Mean age in years (SD)	33.3 (6.0)	33.4 (6.1)	0.96	34.4 (6.5)	34.3 (6.0)	0.83
Hindu (%)	98	95	0.20	95	95	1.00
Caste (among Hindus only) (%):						
-Scheduled caste/tribe (SC/ST) (lowest caste)	14	12	0.61	13	10	0.93
-Other backward caste (socially and educationally disadvantaged)	64	66		68	66	
-General caste	22	22		18	23	
Highest education standard (median, IQR)†	9 (2)	10 (2)	0.211	10 (2)	10 (2)	0.01
**Maternal respondents**	n = 790	n = 769		n = 809	n = 744	
Hindu (%)	91	93	0.09	90	90	0.99
Scheduled caste/tribe (among Hindus only) (%)	51	53	0.38	55	48	0.01
Household Size (median, IQR)†	5 (2)	5 (3)	0.34	5 (2)	5 (3)	<0.01
**Age (years, %):**						
15-19	2	3.0	0.66	7	9	0.18
20-24	42	40		42	44	
25-29	39	41		34	34	
30-34	12	11		13	10	
35-49	6	5		4	4	
Mean age in years (SD)	25.4 (0.2)	25.4 (0.2)	0.78	24.8 (0.2)	25.4 (0.2)	0.04
**Birth parity (%):**						
1 child	32.8	35.1	0.77	38.4	34.8	0.48
2 children	29.6	27.8		27.9	30.5	
3 children	18.7	18.7		17.1	17.2	
4 or more children	18.9	18.3		16.6	17.5	
Ever attended school (%)	75	69	<0.01	63	54	<0.01
Literate (%)	26	32	0.01	33	40	0.01
Below Poverty Line Card (%)	58	57	0.54	73	65	<0.01
**Socioeconomic status quartile (%):‡**						
1	25	22	0.04	20	14	0.01
2	31	28		28	30	
3	27	28		29	33	
4	17	22		23	22	

AWW – Anganwadi Workers, ASHA – Accredited Social Health Activists, ICT-CCS – Information Communication Technology-Continuum of Care Service, IQR – inter-quartile range, SD – standard deviation

*Results at baseline and post-implementation represent two cross-sectional samples. Results are presented without adjusting for survey design. *P*-values reflect results from χ^2^ (categorical variables) or *t* tests (continuous variables), unless otherwise noted; Fisher exact tests were calculated if any cell size for a comparison was <5.

†Wilcoxon rank-sum test used due to non-normal data distribution.

‡Socioeconomic status quartile: Lowest quartile is the poorest. See Methods for further information.

Maternal household respondents were in their mid-20s on average, about 90% were Hindu, and among those who were Hindu about half were in a Scheduled caste (**Table 1**). About one-third of the women sampled had their first child during the year preceding the surveys, median household size was five, half to three-fourths had a BPL card, half to three-fourths had ever attended school but about one-quarter to one-third were literate. School attendance was higher in control than intervention mothers at baseline and post-implementation, whereas literacy levels were higher in intervention than control cohorts for the baseline and post-implementation surveys. In the post-implementation survey, a higher proportion of women in the control than the intervention group had a BPL card.

### ICT-CCS training and use

Based on information collected post-implementation from FLWs in intervention villages about their use of the ICT-CCS phone, nearly all ASHAs and AWWs indicated that they were trained in use of the tool (**Table 2**). Almost two-thirds stated that the most common way they decided who they should visit and who the opposite-cadre FLW should visit was by using the tool. About 85% of ASHAs and AWWs indicated that their ICT-CCS phone was charged and working all or most of the time. Fourteen percent of FLWs said their phone was broken at some time and about three-fourths of FLWs indicated that they had no problem in using the phone. Among the available content, ASHAs and AWWs reported that the videos they showed most commonly were on birth preparedness (68%), followed by newborn care (14%) and family planning (9%). The most common phone-based forms used, other than the home visit scheduler, were to list beneficiaries (46%) and to identify who was due for immunizations (20%). Based on post-implementation reports from maternal respondents in intervention villages, 17% of FLWs used videos on their phone, 16% read lists of questions or reminders from their phones, and 39% used mobile phones with audio during their home visits; the respective percentages reported by maternal respondents in control villages were 0%, 0%, and 25% (all *P* < 0.01).

**Table 2:** ICT-CCS training and usage characteristics reported by front-line workers (FLW) overall and separately for the two cadres – Anganwadi Workers (AWW) and Accredited Social Health Activists (ASHA) – as part of the post-implementation assessment of the ICT-CCS intervention trial in Saharsa, Bihar, 2012-2014

	Overall FLW (n = 316)	AWW (n = 153)	ASHA (n = 163)
Received training on use of ICT-CCS phone from staff who came to village	312 (99%)	151 (99%)	161 (99%)
Used phone before given ICT-CCS phone	258 (82%)	131 (86%)	127 (78%)
**How FLW decides which households to visit herself and which to ask opposite-cadre FLW to visit (missing n = 7):**			
Talk before starting home visit	63 (20%)	29 (19%)	34 (21%)
Inform each other after home visits	18 (6%)	7 (5%)	11 (7%)
Use tool on ICT-CCS phone to communicate progress on home visits	207 (66%)	101 (66%)	106 (65%)
Conduct joint home visits	19 (6%)	11 (7%)	8 (5%)
Other	2 (1%)	0	2 (1%)
**Share of time phone is charged and working:**			
All of the time	180 (57%)	84 (55%)	96 (59%)
Most of the time	92 (30%)	47 (31%)	45 (28%)
Some of the time or less	38 (12%)	16 (11%)	22 (14%)
Mostly discharged	6 (2%)	6 (4%)	0
**Phone ever broken or lost:**			
ICT-CCS phone has broken	43 (14%)	25 (16%)	18 (11%)
ICT-CCS phone had been lost	26 (8%)	16 (11%)	10 (7%)
**Problems faced while using ICT-CCS phone:**			
No problem	233 (74%)	107 (70%)	126 (77%)
Charging battery	17 (5%)	9 (6%)	8 (5%)
No signal or bad signal	53 (17%)	30 (20%)	23 (14%)
Lost information entered	5 (2%)	2 (1%)	3 (2%)
Other	8 (3%)	5 (3%)	3 (2%)
**Videos shown most often on ICT-CCS phone (missing n = 23):**			
Birth preparedness	216 (68%)	104 (68%)	112 (69%)
Newborn care/cord care	44 (14%)	25 (16%)	19 (12%)
Family planning	27 (9%)	14 (9%)	13 (8%)
Complementary feeding	6 (2%)	2 (1%)	4 (3%)
Forms used most often other than home visit scheduler * (missing n = 2):			
Make a list of beneficiaries	146 (46%)	72 (47%)	74 (45%)
Beneficiary management	47 (15%)	21 (14%)	26 (16%)
Immunization due list	62 (20%)	33 (22%)	29 (18%)
Instrument	15 (5%)	9 (6%)	6 (4%)
Growth monitoring	19 (6%)	11 (7%)	8 (5%)
My performance	25 (8%)	7 (5%)	18 (11%)

FLW – Frontline workers, AWW – Anganwadi Workers, ASHA – Accredited Social Health Activists, ICT-CCS – Information Communication Technology-Continuum of Care Service

*The “beneficiary management” form allows FLWs to update beneficiary records. The “growth monitoring” form provides information collected during the last two weighings of all 0-2 y old children in the community. The “my performance” form provides summaries of home visits conducted on time or outstanding, which FLWs could use regularly for planning purposes. The “instrument” form contains a tool to help FLWs determine expected delivery dates, which is needed for registration of pregnant women.

### Impact of ICT-CCS on FLW coordination, confidence and supervision

About half of FLWs asked each other to conduct home visits for them and about two-thirds discussed work together (**Table 3**). For all three questions on this topic, control-intervention differences were significantly greater among AWWs than ASHAs (*P* < 0.10 for the interaction term), with AWWs showing increases and ASHAs showing no differences between intervention and control. FLWs reported that on average they had conducted 1 to 2 home visits with an opposite-cadre FLW during the week prior to the survey; the control-intervention difference was significant only for ASHAs (*P* < 0.01).

**Table 3:** Differences in coordination, job confidence and supervision reported by Anganwadi Workers (AWW) and Accredited Social Health Activists (ASHA) from control vs intervention villages after implementation (July-August, 2014) of the ICT-CCS intervention in Saharsa, Bihar*

	AWW†			ASHA†			*P-*value for difference between AWW and ASHA ‡
**Control (n = 134)**	**Intervention (n = 153)**	***P*-value**	**Control (n = 122)**	**Intervention (n = 163)**	***P*-value**
**Coordination between ASHAs and AWWs:**							
Have you asked an opposite-cadre FLW to conduct a home visit if you were unable to, in the last 30 days) (%)	42	51	0.17	48	41	0.17	0.04
Has an opposite-cadre FLW asked you to conduct a home visit if they were unable to, in the last 30 days (%)	35	47	0.04	47	47	0.91	0.08
Number of home visits conducted jointly with opposite-cadre FLW, in the past 7 days (mean)	1.1	1.2	0.57	1.1	1.9	<0.01	0.14
Met with opposite-cadre FLW to talk about work or home visits in the past 7 days (%)	66	80	0.012	63	62	0.84	0.07
**Job confidence:**							
Feels she has all skills needed for job (%)	28	35	0.15	28	43	<0.01	0.37
**FLW feels she needs skills related to:**							
How to plan home visits (%)	43	28	0.03	48	38	0.17	0.56
How to maintain registers (%)	43	26	0.06	40	30	0.13	0.52
Maternal and newborn health issues (%)	61	62	0.92	64	54	0.20	0.32
How to communicate better with mothers and families (%)	40	55	0.07	53	51	0.82	0.15
**Supervision:**							
Met with supervisor in past 3 months outside sub-center meeting (%)	99	99	0.94	97	97	0.79	0.98
Number of times met with supervisor in past 3 months outside sub-center meeting (mean)	3.5	3.6	0.88	3.9	4.0	0.70	0.90
Supervisor always available by phone or in person when FLW needs to reach her (vs sometimes or never) (%)	77	79	0.59	80	83	0.41	0.83
**During recent visits, supervisor, most of the time:**							
Brought outstanding visits to the FLW’s attention (%)	78	73	0.81	75	73	0.20	0.48
Gave the FLW guidance on what information to give to households (%)	52	55	0.67	56	58	0.82	0.90
Gave the FLW guidance on how to communicate effectively with households (%)	41	47	0.20	38	48	0.16	0.72
Talked to the households the FLW was finding difficult to convince (%)	35	43	0.19	43	43	0.97	0.31
Helped FLW coordinate with her counterpart (%)	50	55	0.26	53	54	0.87	0.58

FLW – Frontline workers, AWW – Anganwadi Workers, ASHA – Accredited Social Health Activists, ICT-CCS – Information Communication Technology-Continuum of Care Service,

*Survey-weighted percentages and counts are reported, to account for the survey design. Regression models were performed that accounted for village as the primary sampling unit and sub-center as the primary stratum within the sampling unit, and with proportional sampling weights at the FLW level. Logistic regression models were conducted for binary outcomes and linear regressions for count variables.

†Separate regression models were conducted for each outcome, for each cadre (AWW, ASHA); *P*-values reflect comparisons of the intervention and control groups, for each cadre.

‡The *P*-values reflect whether treatment effects for each outcome variable differed by cadre and were derived from the following models. To derive these *P* values, we conducted a separate regression model that included all FLWs. These models each contained a term representing cadre (AWW or ASHA), a term representing treatment (intervention or control), and an interaction of these two terms; the *P* values are for these interaction terms and thus reflect whether treatment effects differed by cadre.

Results for measures related to job confidence suggested that overall confidence was higher among FLWs from intervention than control villages but was only significant for ASHAs (*P* < 0.05) (**Table 3**). Confidence related to specific skills varied; results suggested that FLWs from intervention areas tended to be less likely than FLWs from control areas to say they needed more skills related to planning home visits, maintaining registers and, among ASHAs only, on maternal and child health issues. In contrast, more intervention than control AWWs reported a felt need for more skills in communicating with beneficiaries. These differences, however, tended to be non-significant.

Reports on supervision were similar for AWWs and ASHAs and for FLWs from control and intervention villages (**Table 3**). Almost all FLWs in both control and intervention areas reported meeting with their supervisor outside of sub-center meetings in the previous 3 months, and on average met with them approximately monthly. Most FLWs (close to 80%) in both control and intervention areas reported that their supervisor was always available to help by phone or in person. About 70%-80% of FLW workers also reported that their supervisors brought outstanding (ie, due) visits to their attention; half of FLWs reported that supervisors provided guidance on what information to provide and how to communicate it effectively with households; half of FLWs reported that supervisors helped them coordinate with other FLWs; and about 40% of FLWs reported that supervisors interacted with families who were resistant to FLW interactions.

### Impact of ICT-CCS on RMNCHN behaviors

Maternal reports of antenatal visits (3 or more during pregnancy, 2 or more during the last trimester) by FLWs were significantly greater in intervention than control areas post-implementation (*P* ≤ 0.05) (**Table 4**). When baseline levels of last trimester visits were taken into account, there was evidence for an 11 percentage point increase in last-trimester visits attributable to the intervention (*P*-value = 0.04 for the interaction term). Maternal tetanus toxoid injections and use of iron-folic acid tablets tended to be higher among women from intervention than control villages, but there was no evidence of differences being attributable to the intervention.

**Table 4:** Differences attributable to the ICT-CCS intervention on selected indicators reported by maternal household respondents as part of the ICT-CCS intervention trial in Saharsa, Bihar 2012-2014*

	Baseline† (May-June 2012)			Post-implementation† (July-August 2014)			Percent difference attributable to ICT-CCS‡	*P*-value
**Modeled outcome**	**Control**	**Intervention**	***P*-value**	**Control**	**Intervention**	***P*-value**
**Antenatal care:**								
At least 3 antenatal home visits (%)	24	37	<0.01	29	48	0.05	6.6	0.31
2 or more home visits in last trimester (%)	36	35	0.83	42	51	<0.01	10.7	0.04
At least 2 tetanus toxoid injections (%)	94	95	0.66	89	94	0.05	3.8	0.22
Consumed at least 90 iron-folic acid tablets (%)	10	15	0.03	11	17	<0.01	0.4	1.00
Received iron-folic acid tablets by month 4 (%)	22	28	0.09	15	20	<0.01	-0.9	0.89
**Home visits after delivery:**								
At least one home visit within 24 h of delivery, among women who had a home delivery (%)	N/A	N/A	N/A	35	34	0.90	N/A	N/A
Any visit in the first week (%)	N/A	N/A	N/A	60	72	<0.01	N/A	N/A
Any visit after first week but before first month (ie, weeks 2-4) (%)	N/A	N/A	N/A	45	48	0.10	N/A	N/A
Total number of home visits in the first month (mean) (%)	N/A	N/A	N/A	1.8	2.1	0.14	N/A	N/A
**Delivery and newborn care:**								
Facility delivery (%)	77	76	0.80	84	85	0.93	2.1	0.65
Nothing applied to the umbilical cord (%)	26	23	0.22	33	31	0.69	2.4	0.53
Bath delayed by at least 2 days (%)	45	41	0.26	48	44	0.33	-0.10	0.90
Skin-to-skin care (%)	25	17	0.03	58	63	0.02	13.4	<0.01
Immediate breastfeeding (within 1 hours of delivery) (%)	47	44	0.58	62	74	<0.01	14.7	<0.01
**Exclusive breastfeeding:**								
Exclusive breastfeeding in past 24 h, among infants <6 months old (%)§	64	60	0.23	70	67	0.73	1.7	0.67
Exclusive breastfeeding for first 6 months, among infants ≥6 months old (%)	38	34	0.25	61	62	0.63	4.5	0.31
**Complementary feeding, among infants ≥6 months old:**								
Any home visit related to complementary feeding (%)	1	2	0.11	37	46	0.02	8.3	0.62
Eats solid or semisolid food (%)	64	57	0.17	55	63	0.03	16.2	0.01
Began eating solid food by age 6 months (%)	52	39	0.02	32	39	0.06	20.8	<0.01
Fed solid/semisolid food in previous day (%)	53	50	0.65	51	58	0.04	10.8	0.11
Appropriate frequency of cereal-based feedings (%)‖	27	31	0.26	32	39	0.10	2.6	0.79
**Immunizations, among infants ≥6 months old:**¶								
Received DPT3 (%)	63	65	0.62	77	79	0.96	0	0.97
Fully immunized (except measles) (%)	40	41	0.75	55	59	0.51	3.4	0.68
**Family planning and reproductive health:**								
Any home visit about family planning or postpartum health (%)**	14	12	0.39	27	30	0.26	6.6	0.17
Current use of temporary methods of contraception (child age ≥6months) (%)†	8	8	0.80	11	10	0.49	2.8	0.55
Current use of any modern method of contraception (%)‡‡	18	19	0.82	28	35	0.03	6.5	0.11

N/A – not available, ICT-CCS – ICT-CCS – Information Communication Technology-Continuum of Care Service, DPT3 – diptheria-pertussis-tetanus vaccine

*Analyses of all women included a maximum of 790 women from control and 769 from intervention villages at baseline, and 809 from control and 744 from intervention villages at post-implementation The respective maximum numbers of women with an infant <6 months old were 341, 344, 302 and 291. The respective numbers of women with an infant ≥6 months old were 417, 399, 490, 437. Table S1 in **Online Supplementary Document** shows the exact number of maternal respondents from whom data was available for each outcome. In **Table 3**, survey-weighted percentages and counts are reported, to account for the survey design. Regression models were performed that accounted for village as the primary sampling unit and sub-center as the primary stratum within the sampling unit, with proportional sampling weights at the maternal respondent/household level. All models were adjusted for maternal age, household size, whether a woman belonged to a scheduled caste or tribe, literacy, lack of formal education, having a Below Poverty Line card, and socioeconomic status (SES) quartile.

†Separate regression models were conducted for each outcome, at baseline and post-implementation; *P*-values reflect comparisons of the intervention and control groups, at each time point.

‡In order to estimate the effect of the intervention on a particular outcome, we conducted a separate regression model for each outcome that included all maternal respondents. These models each contained a term representing time (baseline or post-implementation), a term representing treatment (intervention or control), and an interaction of these two terms, which is represented by the difference in difference estimator (DID) and its *P* value. The DID reflects treatment effects (positive values reflect the amount of improvement attributable to the intervention).

§Based on reports of liquids and solids fed to children younger than 6 months in the previous 24 h, following the recommended definition of the World Health Organization.

‖Defined as 2 or more times for children 6-8 months of age and 3 or more times for children 9-11 months of age.

¶Vaccination was reported based on immunization card or self-report.

**Includes discussions on excessive vaginal bleeding, severe pain in lower abdomen, high fever, and foul-smelling vaginal discharge.

††Defined as use of birth control pills, condoms, injectables, or an IUD.

‡‡Defined as use of male or female sterilization, birth control pills, condoms, injectables, or an IUD.

The proportion of home-born newborns who were visited at home within 24 hours (about one-third) was no different in intervention vs control areas (**Table 4**). However, the proportion of newborns who had home visits during the first week after delivery (72% vs 60%, *P* < 0.01) was significantly higher in intervention than control areas post-implementation, although attribution to the intervention is uncertain given the lack of baseline data. Increases in the proportions of newborns in the intervention vs the control areas who were visited in the late neonatal period (weeks 2-4) (48% vs 45%, respectively, *P* = 0.10) and the total number of visits by a FLW in the first month (2.1 vs 1.8, respectively, *P* = 0.14) did not reach statistical significance. The intervention resulted in a significant increase in the practice of skin-to-skin care (13 percentage point increase attributable to the intervention, *P* < 0.01) and the frequency of breastfeeding immediately after delivery (15 percentage point increase attributable to the intervention, *P* < 0.01). There were no significant differences attributable to the intervention in the other behaviors related to delivery and newborn care, including facility delivery, cord care, delay of bathing, or exclusive breastfeeding, although it is notable that these behaviors were higher at post-implementation than at baseline, among women from both control and intervention villages.

Home visits related to complementary feeding were relatively rare at baseline (1%-2%) but significantly higher in intervention vs control areas post-implementation (46% vs 37%, *P* = 0.02); no difference, however, could be attributed to the intervention. Increases of 16% for eating solid or semisolid foods and 21% for the introduction of solid food by 6 months were attributable to the intervention (*P* values ≤0.01), although declines in these two desired behaviors among control women contributed to these differences. There were no significant increases in infant immunizations attributable to the intervention. Home visits for family planning or postpartum health were higher post-implementation than at baseline in both the control (27% vs 14%, respectively) and intervention areas (30% vs 12%, respectively), however, the 6 percentage point increase attributable to the intervention did not reach statisitical significance (*P* = 0.17). Current use of temporary methods of family planning was unchanged, but use of any modern method of contraception was higher post-implementation than at baseline in both the control (28% vs 18%, respectively) and intervention areas (35% vs 19%, respectively), and the 6 percentage point increase attributable to the intervention nearly reached statisitical significance (*P* = 0.11).

## DISCUSSION

This study evaluated the impact of a mHealth tool on FLW coordination, confidence and supervision, as well as on RMNCHN-related behaviors among women who had given birth in the previous year in rural Bihar, India. Training of FLWs on use of the phone was rigorous, the phones worked most of the time, most FLWs reported no problems in using the phones, and most FLWs reported that the ICT-CCS was their most important tool for home visitation planning. The intervention was also associated with more coordination of AWWs with ASHAs and increased FLW job confidence, primarily among ASHAs. However, supervision of AWWs and ASHAs was unaffected, perhaps because the supervisory module was introduced relatively late in the implementation phase of the study. After accounting for baseline differences between women from the intervention and control villages, we found significant increases in home visits in the late antenatal period, and in the uptake of skin-to-skin care, breastfeeding immediately after delivery, and age-appropriate complementary feeding. The increased use of any modern method of contraception was borderline significant, and early postnatal home visits were higher in the intervention compared to the control areas in the post-implementation phase, but unfortunately lack of baseline data for this analysis precluded attribution of the difference to the impact of the intervention. The majority of targeted RMNCHN behaviors, however, were not significantly impacted. Nevertheless, the behaviors that were improved have powerful potential to improve infant health and survival [17-19].

These results corroborate extensive previous global evidence [19-23] as well as findings from neighborhing Uttar Pradesh [24-28] – a state which shares many sociodemographic and health system characteristics with Bihar – demonstrating that increasing the number and quality of FLW interactions with beneficiaires through outreach and home visitation can improve a variety of RMNCHN outcomes, including skin-to-skin care, immediate breastfeeding and complementary feeding. Our study extends these findings, however, and shows that FLW reach to beneficiaries through home visitation and their effectiveness in advancing the adoption of health-promoting and life-saving RMNCHN practices can be increased through use of the ICT-CCS mHealth tool. Systematic reviews suggest that mHealth tools may result in improvements in a variety of aspects of FLW work activities, such as case management, data collection, development of support networks, and adherence to recommendations regarding treatment and advice [3,20,21]. Although the use of mHealth tools is increasing, few other rigorously designed evaluations of their effectiveness in highly resource-constrained settings exist. Some prior mHealth interventions involving use of multi-facted phone-based applications by FLWs to improve RMNCHN do exist. Interventions in rural Tanzania and the state of Jharkhand, India (the latter involved ASHAs) reported that the interventions resulted in increased facility deliveries [22,23]. An mHealth intervention for ASHAs in the state of Uttar Pradesh did not observe effects on facility delivery, but did observe improvements in maternal use of iron-folic acid tablets and report of complications before and after delivery, which was likely related to improved knowledge [24].

It is challenging to compare our results directly to prior studies. The ICT-CCS tool was complex; it facilitated a variety of FLW activities from scheduling home visits to improving their content, and it targeted a wide range of RMNCHN-related topics. The tool was also implemented in the context of *Ananya*, which was itself a unique and multi-faceted program. A prior evaluation of the ICT-CCS intervention compared the frequency of maternal RMNCHN-related behaviors in the intervention group, based on monitoring data routinely collected from their phones, to survey data for the rest of Bihar that were collected by the National Rural Health Mission in 2012-13 [25]. Based on this design, the study was unable to differentiate the effects of *Ananya* and the ICT-CCS intervention, and thus the contribution of ICT-CCS to the observed improvements could not be determined. Our study was able to overcome this limitation, demonstrating that even in the context of the *Ananya* program, further benefits to RMNCHN could be achieved through use of the ICT-CCS tool.

Despite the improvements observed through use of the ICT-CCS tool, there are several potential reasons why more significant changes in a wider range of RMNCHN behaviors were not observed. One reason could be the limitations that remained in FLW coordination, confidence and supervision. It appears that the ASHAs continued to view their work as relatively independent from the AWWs. This may be related to the observation that ASHAs gained confidence through use of ICT-CCS whereas AWWs did not. Moreover, more AWWs (but not ASHAs) in intervention than control areas reported a felt need for more skills in communicating with beneficiaries, which may reflect the increased exposure to beneficiaries in home settings and a greater self-awareness among AWWs of the challenges of influencing behavior change in these settings. These data may also reflect an emphasis within the *Ananya* program on newborn care, which had primarily been the responsibility of ASHAs pre-*Ananya*. However, as *Ananya* sought to equalize their roles, more shift in tasks may have been expected of the AWWs than the ASHAs, which may explain why the AWWs sought to coordinate more with the ASHAs than vice versa. The health administration structure may also have affected impact; for example, the fact that ASHAs and AWWs were managed by two different government ministries could have been a structural barrier to coordination among them that could not be overcome by the ICT-CCS tool. However, the programs shared a broad set of objectives which were among the primary targets for the intervention. Nevertheless, the FLWs likely encountered significant challenges in shifting roles and in coordinating on shared objectives. While the potential for community-based health workers to improve RMNCHN outcomes is vast, their impact is limited by the health systems in which they work [26,27]. Systemic challenges include expectations of FLWs to cover large underlying populations and imbalances between hours worked and incentives [28]. However, it is also important to note that implementation of a tool like ICT-CCS may itself strengthen the health system, through improving FLW effectiveness, efficiency, knowledge and motivation [25]. It is possible that implementation in the context of *Ananya,* and moreover, in the context of dysfunctional systems in Bihar reduced the impact of the ICT-CCS intervention, but this cannot be determined. Since one of the important functions of the tool was to facilitate visit reminders and reporting, the parallel continuation of legacy paper/register-based Management Information Systems could have added to the ‘burden’ this tool might have placed on FLW workload. It is also noteworthy that some behaviors improved among women from control and intervention villages, which is likely at least in part a function of the *Ananya* program. For example, skin-to-skin care and immediate breastfeeding improved substantially in both groups, but improvements were significantly greater among women from the intervention villages. It is possible that impact of an ICT-CCS type intervention would be greater if implemented independently of the context of a program like *Ananya*.

As observed recently in neighboring Uttar Pradesh, facilitation of adherence to protocols through implementation of tools like the ICT-CCS may be insufficient to improve maternal and child health outcomes in the absence of system-wide shifts in incentives and improvements in skills in communicating with families and managing behavior change [20,21,29,30].

It is also possible that, although the FLWs received extensive training on use of the tool and technical support, it may not have been adequate. We do not have information about the extent of the FLWs’ understanding of the basic health messages they were asked to impart or the quality of their delivery of these messages during home visits. Prior studies of ASHA performance in Indian states other than Bihar concluded that improved FLW training was needed in general [31,32]. It is also uncertain why some behaviors were impacted but not others. Impact may have been greater on some behaviors vs others due to program emphasis on certain behaviors. For example, *Ananya* emphasised newborn care, and we indeed observed significant increases in skin-to-skin care and immediate breastfeeding. Other explanations may also contribute to observed impacts. For example, impact may have been minimal on consumption of iron-folic acid tablets because of limited supply, and on family planning because of strong social norms. We do not know why some complementary feeding behaviors declined in control villages.

Beyond this study, the ICT-CCS tool has been a successful *Ananya* intervention in terms of its adoption and scale-up by the Government of India. Evidence from this study directly informed the decision of the government to roll out an adapted version of the software with the aim to reach more than 100 000 AWWs across eight prioritized states – Bihar, Uttar Pradesh, Madhya Pradesh, Maharashtra, Rajasthan, Andhra Pradesh, Chattisgarh and Jharkhand – with plans to scale-up across all 29 states after the initial rollout. This initiative is being driven through the ICDS, which modified the tool because the original tool was designed specifically for both ASHAs and AWWs, and the current scale-up effort is only for AWWs. Additionally, the ICT software is a key component of the implementation of the National Nutrition Mission (NNM), a joint effort between the MWCD, the MoHFW, and the Ministry of Drinking Water and Sanitation, with the overarching goal of reducing stunting and undernutrition at a national scale. The software application is being further customized for this rollout and links to a comprehensive web-based dashboard, providing real time information about ICDS service delivery and gradually phasing out the current widespread use of paper registers. Given the extent to which the ICT-CCS tool has been adapted and scaled by various ministries, going forward it appears to be a significant component of efforts to strengthen FLW “last mile” service provision and monitoring.

A key strength of this analysis is its cluster randomized trial design, which facilitates the ability to attribute changes to the intervention. In particular, we were able to conduct a DID analysis, which incorporates adjustment for changes in the measured outcomes from baseline to post-implementation. This was particularly important given that the intervention was conducted in the context of the *Ananya* program, which was expected to impact the same RMNCHN-related behaviors as the ICT-CCS intervention. We acknowledge, however, that the DID approach has more limited power than a post-only comparison [33], which was the basis of power calculations that were conducted for the original study design. The evaluation design was a repeated cross-section rather than repeated interviews with the same women. The approach to sampling likely improved the representativeness of the study respondents (and accordingly minimized selection bias) and reduced logistical challenges but does not allow control for fixed effects at the individual or household level. However, we did adjust for differences in sociodemographic variables in each sample; these adjustments were important but did not substantially impact the final results (data not shown). Another strength was the availability of information from FLWs related to their uptake of the intervention, which aids in assessing whether lack of impact was due to limitations in use or efficacy of the tool.

A limitation of this study is that some information was not available at both evaluation time points (eg, certain variables collected from FLWs, and maternal reports of frequency of home visits for newborns). In addition, information on actual health outcomes was not available, such as maternal or infant morbidities or mortality; collection of this type of information was not deemed feasible. Supervision was an important aspect of the intervention, but limited information was collected from FLW supervisors. We relied primarily on self-reported data from the FLWs and maternal respondents; data were not available to validate these reports. We expect reporting bias may be minimal in this study context, however, given that everyone was exposed to the *Ananya* program, which was also trying to improve FLW effectiveness and RMNCHN-related behaviors. The impact of the tool on equity requires further examination.

In summary, this large-scale implementation of a multi-faceted mHealth tool suggests that it had positive impacts on some health-related behaviors that are linked to improved infant health and survival [17,19,26,27]. Modest impacts on FLW coordination and confidence may have contributed to these impacts. Challenges that may have limited the tool’s impact include potential under-utilization (the tool encompassed a variety of features) and factors related to the existing health system infrastructure. Greater programmatic focus on behavior change management may be needed in order to realize greater RMNCHN impacts in conjunction with use of the tool. Despite these challenges, FLWs readily adopted the tool, and health behavior impacts were achieved, which bodes well for future programs focused on implementing mHealth tools in similar settings. This evidence helps to inform new national and global strategies for advancing RMNCHN through mHealth technology.

## Additional material

Online Supplementary Document
