# Supplementary material for: Use of mobile technology by frontline health workers to promote reproductive, maternal, newborn and child health and nutrition: a cluster randomized controlled Trial in Bihar, India
Source: J Glob Health. 2019 Nov 24;9(2):020424. doi: 10.7189/jogh.09.020424 (PMC6875677; doi:10.7189/jogh.09.020424)
Supplement: Online Supplementary Document [file jogh-09-020424-s001.pdf]

SUPPLEMENTARY MATERIALS

Supplementary Table 1: Number of subjects with data on each outcome.

	Baseline (May-June 2012)		Post-implemen-tation (July-August 2014)	
Modeled outcome	Contr ol	Inter-vention	Control	Inter-vention
**Antenatal Care**				
At least 3 antenatal home visits (%)	790	769	809	744
2 or more home visits in last trimester (%)	790	768	809	744
At least 2 tetanus toxoid injections (%)	790	769	808	744
Consumed at least 90 iron-folic acid tablets (%)	790	769	809	744
Received iron-folic acid tablets by month 4 (%)	790	769	809	744
**Home Visits After Delivery**				
Visit in first 24 Hours (%)	n.a.	n.a.	806	743
In the home delivered subset: Visit in first 24 Hours (%)	n.a.	n.a.	152	111
Any visit in the first week (%)	n.a.	n.a.	793	735
Any visit after first week but before first month (%)	n.a.	n.a.	795	737
Total number of home visits in the first month (mean) (%)	n.a.	n.a.	809	744
**Delivery and Newborn Care**				
Facility delivery (%)	789	767	809	741
Nothing applied to the umbilical cord (%)	790	769	774	706
Bath delayed by at least 2 days (%)	758	743	781	719
Skin-to-skin care (%)	790	769	809	743
Immediate breastfeeding (within 1 hour of delivery) (%)	790	769	809	744
**Exclusive Breastfeeding**				
Exclusive breastfeeding in past 24 hours, among infants <6 months old (%) [d]	273	309	259	248
Exclusive breastfeeding for first 6 months, among infants ≥6 months old (%)	417	399	486	433
**Complementary feeding, among infants ≥6 months old**				
Any home visit related to complementary feeding (%)	401	386	490	437
Eats solid or semisolid food (%)	417	399	490	437
Began eating solid food by age 6 months (%)	417	399	490	437
Fed solid/semisolid food in previous day (%)	415	397	488	433

Appropriate frequency of cereal-based feedings (%) [e]	417	399	490	437
**Immunizations, among infants ≥6 months old**				
Received DPT3 (%)	416	394	490	437
Fully immunized (except measles) (%)	416	394	490	437
**Family Planning and Reproductive Health**				
Any home visit about family planning or postpartum health (%) [f]	787	768	809	744
Current use of temporary methods of contraception (child age ≥6mo) (%) [g]	408	392	476	423
Current use of any modern method of contraception (%) [h]	773	754	788	712

<u>Statistical Appendix</u>
"Use of Mobile Technology by Frontline Health Workers to Promote Reproductive, Maternal, Newborn and Child Health and Nutrition: A Cluster Randomized Controlled Trial in Bihar, India"

Appendix prepared by Stanford Ananya Study Data team

Table 1.
Demographics characteristics of FLWs (ANMs, ASHAs and AWWs) as well as maternal respondents were compared for all sampled respondents in the ICT-CCS trial according to the baseline and post-implementation groups. Percentages were reported as crude percentages without adjusting for any survey design or weights. P values were calculated using two-sample t-test for the continuous variables and chi-square test for the categorical variables. If the sociodemographic characteristic represented in rows did not fit the normality assumption (was invalidated), median and inter-quantile range (IQR) was reported instead of mean and standard deviation and Mann-Whitney U-Test was performed. Fisher's-exact test was conducted for categorical variables with cellular frequency of less than 5 in any sub-group.

Table 2.
This table reported training and usage characteristics reported by FLWs overall and separately for the two cadres – AWWs and ASHAs. As these questions were only available post-intervention, we simply reported percentages and no further statistical comparisons were made. Percentages were reported as crude percentages without adjusting for any survey design or weights. Analyses were conducted in STATA version 14.

Table 3.
Differences in coordination, job confidence and supervision reported by AWWs and ASHAs from control versus intervention villages after implementation (July-August, 2014) of the ICT-CCS intervention in Saharsa, Bihar.
For all the indicators related to coordination between cadres, job confidence and supervision in this table, we set up our survey design using village as the primary sampling unit, and subcenter as stratum with sampling weights at the FLW level. Our analytical cohort in this table was responses from the survey data for the AWWs and ASHAs at the end of the trial. To compare the difference of the responses between intervention and control groups for both AWWs and ASHAs, survey logistic regressions were performed to obtain the p-value for the difference comparing the intervention vs the control without adjusting for any participant-level covariates. For each binary indicator, we also fit a separate survey logistic regression with all our AWW and ASHA participants to get the p-value of the difference in difference estimator (DID, interaction between FLW type and treatment group) without adjusting for any participant-level covariates. Specifically, the independent variables in the logistic regression for DID included treatment group, FLW group and the interaction term of the two. The dependent variable was our binary indicator of interest. To calculate percent difference between ASHA &

AWW in terms of the treatment effects, we predicted the probability of the indicator via our regression and computed the DID in probabilities of the indicator between the treatment and control group between ASHAs and AWWs. For discrete counts such as ‘Number of home visits conducted jointly with opposite-cadre FLW, in the past 7 days’, survey linear regressions were conducted treating outcome indicators as continuous variables instead, using the same survey design set-up as survey logistic regressions. In addition, we reported average difference in visits/times between ASHAs & AWWs directly from the coefficient in the interaction term in survey linear regression. All analyses were conducted by using the Survey procedures in Analyses and were conducted in SAS version 9.4 (Surveyreg surveylogistic) and STATA version 14 (svyreg svylogit).

Table 4.

Differences attributable to the ICT-CCS intervention on selected indicators reported by maternal household respondents as part of the ICT-CCS intervention trial in Saharsa, Bihar 2012-2014

To evaluate the impact of the ICT-CCS intervention on selected maternal and child health indicators, we utilized survey sampling weights according to this survey’s design. Village was the primary sampling unit, sub-center was the stratum and sample weights were calculated at the household level. Our analytical cohort in this table was responses from the survey data for all maternal respondents pre- and post-implementation of the trial. Rates of occurrence of all our binary indicators were reported by the intervention groups (intervention vs control) using survey commands in SAS/STATA to account for the survey design. To compare the difference of the responses between intervention and control group, survey logistic regressions or survey linear regression were performed to get the p-value for the difference in indicator comparing the treatment vs the control adjusting for selected maternal respondents’ demographics variables. This approach was conducted separately for the pre-intervention period only, post-implementation period only and then the last set included a difference in difference estimator (DID, interaction between intervention group and time) including data from both the pre- and post-implementation periods simultaneously. In the last set, the DID was calculated. All models were calculated adjusting for selected maternal respondents’ demographics variables. Models were calculated using the Survey procedures in Analyses were conducted in SAS version 9.4 (Surveyreg surveylogistic) and STATA version 14 (svyreg svylogit).

Reference:

1. Kish KL "Survey Sampling” : Kish, Leslie (1995) Survey Sampling, Wiley, ISBN 0-471-10949-5
2. SAS. Survey procedures: Lewis, Taylor H. Complex Survey Data Analysis with SAS. CRC Press, 2016.
3. Survey Data Manual: A Stata Press Publication (2017), ISBN13: 978-1-59718-252-2

Appendix. List of Variables for reproducibility

Table 1. Demographic characteristics of Anganwadi Workers (AWW), Accredited Social Health Activists (ASHA), and maternal household respondents as part of the ICT-CCS intervention trial in Saharsa, Bihar, 2012-2014. [a]

Original Variable	Any Modification (R Codes)	Description
endline		Endline (Y/N)<br>0: Baseline<br>1: Endline (Post-implementation in table)
Treatment		Treatment group (y/n)
ASHA or AWW (ASHA_AWW_ICT_RUF.DTA)		
asha or aww		Asha (y/n)<br>0: AWW<br>1: ASHA<br>Or AWW(Y/N)<br>0:ASHA<br>1:AWW
t0_a1 and t1_a01		Live in the village that provided service (y/n)
t0_A6 and t1_A06		- Age (continuous)
t0_a7 and t1_a07		- Hindu (y/n)
t0_a9 and t1_a9	T0_a7=**1 and** t0_a9 for endline=0<br>T1_a7=**1 and** t1_a9 for endline=1	Caste (for Hindu Only)<br>- Scheduled caste<br>- -Scheduled tribe<br>- Other backward class<br>- General Class<br>- Other(Specify)
t0_a12 and t1_a13		Highest grade of education (Continuous)
Maternal Respondents (file:ICT_RCT_HH_RUF.DTA)		
t0_hh_hindu and t1_hh_hindu		Hindu (y/n)
t0_hh_scst and t1_hh_scst		Caste (for Hindu Only)<br>- Scheduled caste<br>- -Scheduled tribe<br>- Other backward class<br>- General Class<br>Other(Specify)
t0_b02 and t1_hh_sizehh		Household Size
t0_hh_agefml_cat_rk from t0_hh_agefml<br><br>and<br>t1_hh_agefml_cat	t0_hh_agefml_cat_rk=.;<br>if 14<t0_hh_agefml<20 then t0_hh_agefml_cat_rk=1;<br>else if 19<t0_hh_agefml<25 then t0_hh_agefml_cat_rk=2;	Age category

	else if 24<t0_hh_agefml<30 then<br>t0_hh_agefml_cat_rk=3;<br>else if 29<t0_hh_agefml<35 then<br>t0_hh_agefml_cat_rk=4;<br>else if 34<t0_hh_agefml<50 then<br>t0_hh_agefml_cat_rk=5;	
t0_hh_agefml		Age
t0_hh_numkid_cat_rk from<br>t0_hh_numkid<br>and<br>t1_hh_numkid_cat	t0_hh_numkid_cat_rk=.;<br>if t0_hh_numkid=1 then<br>t0_hh_numkid_cat_rk=1;<br>else if t0_hh_numkid=2 then<br>t0_hh_numkid_cat_rk=2;<br>else if t0_hh_numkid=3 then<br>t0_hh_numkid_cat_rk=3;<br>else if t0_hh_numkid>3 then<br>t0_hh_numkid_cat_rk=4;	Birth Parity
t0_hh_educ and t1_hh_educ		Ever Attended school (0/1)
t0_hh_literate and<br>t1_hh_literate		Literate (0/1)
t0_hh_bpl and t1_hh_bpl		Below poverty line (0/1)
t0_hh_wealthindex_qurt and<br>t1_hh_wealthindex_qurt		Socio-economic status<br>(Quartile of<br>t0_hh_wealthindex)

Table 2: ICT-CCS training and usage characteristics reported by front-line workers (FLW) overall and separately for the two cadres – Anganwadi Workers (AWW) and Accredited Social Health Activists (ASHA) – as part of the post-implementation assessment of the ICT-CCS intervention trial in Saharsa, Bihar, 2012-2014.

Original Variable	Any Modification (R Codes)	Description
eflw_phn_trng		Received training on use of ICT-CCS phone from staff who came to village
eflw_phn_used_past		Used phone before given ICT-CCS phone
t1_d18		How FLW decides which households to visit herself and which to ask opposite-cadre FLW to visit
t1_d07		Share of time phone Is charged and working
eflw_phn_damage		ICT-CCS phone has broken

t1_d08_1		ICT-CCS phone had been lost
t1_d12_1		Problems faced while using ICT-CCS phone
t1_d39_1		Videos shown most often on ICT-CCS phone
t1_d36_1		Forms used most often other than home visit scheduler

Table 3. Differences in coordination, job confidence and supervision reported by Anganwadi Workers (AWW) and Accredited Social Health Activists (ASHA) from control versus intervention villages after implementation (July-August, 2014) of the ICT-CCS intervention in Saharsa, Bihar.

Original Variable	Any Modification (R Codes)	Description
t1_c77 = how many times in the past 30 days have you asked the aww to cover a home … t1_c76= have you ever asked the aww to help you by conducting a home visit when you	if t1_c77>0 then askedhelpin30=1; else if t1_c77=0 then askedhelpin30=0; else if t1_c76=0 then askedhelpin30=0;	Have you ever asked an opposite cadre FLW to conduct a home visit if you were unable to (in last 30 days?) (%)
t1_c78 has the aww ever asked you to help her by conducting a home visit when she .. t1_c79 = how many times in the past 30 days has the aww asked you to cover a home..	if t1_c79>0 then flwaskedhelpin30=1; else if t1_c79=0 then flwaskedhelpin30=0; else if t1_c78=0 then flwaskedhelpin30=0	Has opposite cadre FLW ever asked you to conduct a home visit if they were unable to (in the last 30 days) (%)
t1_c83 in the past 7 days did you do any home visits jointly with the aww? …	.	Number of home visits conducted jointly with opposite-cadre FLW in the past 7 days (mean)
t1_c80 =how many times did you meet with the aww in the past 7 days to talk about ..	if t1_c80>0 then talkabtwrk=1; else if t1_c80=0 then talkabtwrk=0;	Met with opposite-cadre FLW to talk about work or home visits in the past 7 days (%)
t1_e15	.	Feels she has all skills needed for job
.	.	FLW feels she needs skills related to:
t1_e16_code1	.	How to plan home visits
t1_e16_code2	.	How to maintain registers

t1_e16_code3	.	Maternal and newborn health issues
t1_e16_code4	.	How to communicate better with mothers and families
T1_e2 in the last 3 calendar months, did you meet your [supervisor] not counting…	.	Met with supervisor in past 3 months outside sub-center meeting (%)
T1_e04: how many times did you meet your [supervisor] in the last 3 calendar months	.	Number of times met with supervisor in past 3 months outside sub-center meeting (mean)
.	.	Supervisor available by phone or in person when FLW needs to reach her (%):
eflw_sup_alwaysavail	.	Always
eflw_sup_someavail	.	Sometimes
eflw_sup_neveravail	.	Never
	.	During recent visits, supervisor, most of the time:
eflw_outst_vis	.	Brought outstanding visits to the FLW's attention (%)
eflw_info_hh	.	Gave the FLW guidance on what information to give to households (%)
eflw_comm_hh	.	Gave the FLW guidance on how to communicate effectively with households (%)
eflw_conv_hh	.	Talked to the households the FLW was finding difficult to convince (%)
eflw_coord	.	Helped FLW coordinate with her counterpart (%)

Table 4. Differences attributable to the ICT-CCS intervention on selected indicators reported by maternal household respondents as part of the ICT-CCS intervention trial in Saharsa, Bihar 2012-2014.

Original Variable	Any Modification (R Codes)	Description
Modeled outcome		
anc_atleast3visits	max(t0_hh_anc_atleast3visits,t1_hh_anc_atleast3visits	At least 3 antenatal home visits
flw_visit_tot_3trim_atl2	max(t0_hh_flw_visit_tot_3trim_atl2,t1_hh_flw_visit_tot_3trim_atl2	2 or more home visits in last trimester
anc_tt_atleast2inj	max(t0_hh_anc_tt_atleast2inj, t1_hh_anc_tt_atleast2inj	At least 2 tetanus toxoid injections
anc_ifa_atl90con	max(t0_hh_anc_ifa_atl90con, t1_hh_anc_ifa_atl90con	Consumed at least 90 iron-folic acid tablets
anc_ifa_atl90rec	max(t0_hh_anc_ifa_atl90rec, t1_hh_anc_ifa_atl90rec	Received iron-folic acid tablets by month 4
t1_hh_flw_visit_first24	.	Visit in first 24 Hours
t1_hh_flw_visit_firstweek	.	Any visit in the first week

t1_hh_flw_visit_1st month_not1stw	.	Any visit after first week but before first month
t1_hh_flw_visit_tot1 stmnth	.	Total number of home visits in the first month (mean)
dppc_fac_del	max(t0_hh_dppc_fac_del,t1_hh_dppc_f ac_del	Facility delivery
dppc_nothingapplied	max(t0_hh_dppc_nothingapplied,t1_hh_ dppc_nothingapplied	Nothing applied to the umbilical cord
dppc_bathdelayedatl 2	max(t0_hh_dppc_bathdelayedatl2 ,t1_hh_dppc_bathdelayedatl2	Bath delayed by at least 2 days
dppc_skintoskin	max(t0_hh_dppc_skintoskin ,t1_hh_dppc_skintoskin	Skin-to-skin care
dppc_immbrfeed	max(t0_hh_dppc_immbrfeed ,t1_hh_dppc_immbrfeed	Immediate breastfeeding (within 1 hour of delivery)
dppc_excbrfeedpast 24	max(t0_hh_dppc_excbrfeedpast24 ,t1_hh_dppc_excbrfeedpast24	Exclusive breastfeeding in past 24 hours, among infants <6 months old b
dppc_excbrfeed6mth	max(t0_hh_dppc_excbrfeed6mth,t1_hh_ dppc_excbrfeed6mth	Exclusive breastfeeding for first 6 months, among infants ≥6 months old
flw_visit_compfeed	max(t0_hh_flw_visit_compfeed ,t1_hh_flw_visit_compfeed	Any home visit related to complementary feeding
feed_eat_solidsemis	max(t0_hh_feed_eat_solidsemis ,t1_hh_feed_eat_solidsemis	Eats solid or semisolid food
feed_solid_by6mth	max(t0_hh_feed_solid_by6mth ,t1_hh_feed_solid_by6mth	Began eating solid food by age 6 months
feed_anycereal_prev day	max(t0_hh_feed_anycereal_prevday,t1_ hh_feed_anycereal_prevday	Fed solid/semisolid food in previous day
feed_approp_frq	max(t0_hh_feed_approp_qty ,t1_hh_feed_approp_frq	Appropriate frequency of cereal-based feedings c
imm_dpt3by6_card	max(t0_hh_imm_dpt3by6_card ,t1_hh_imm_dpt3by6_card	Received DPT3
imm_fullimm_card	max(t0_hh_imm_fullimm_card ,t1_hh_imm_fullimm_card	Fully immunized (except measles)
flw_visit_flyplan	max(t0_hh_flw_visit_flyplan ,t1_hh_flw_visit_flyplan	Any home visit about family planning or postpartum health d
fp_contr_temp_r	max(t0_hh_fp_contr_temp_r ,t1_hh_fp_contr_temp_r	Current use of temporary methods of contraception (child age >6mo) e
fp_contr_anymodern _r	max(t0_hh_fp_contr_anymodern_r ,t1_hh_fp_contr_anymodern_r	Current use of any modern method of contraception f
Covariates		
hh_agefml	max(t0_hh_agefml,t1_hh_agefml);	Age
hh_scst	max(t0_hh_scst,t1_hh_scst);	SCST category
hh_literate	max(t0_hh_literate,t1_hh_literate);	literate
hh_educ	max(t0_hh_educ,t1_hh_educ);	education
hh_wealthindex	max(t0_hh_wealthindex,t1_hh_wealthin dex);	wealth index
hh_sizehh	max(t0_b02,t1_b02);	household size
hh_bpl	max(t0_hh_bpl,t1_hh_bpl);	below poverty line

hh_rel	if t0_hh_hindu=0 then t0_hh_rel=2;else t0_hh_rel=t0_hh_scst;<br>if t1_hh_hindu=0 then t1_hh_rel=2;else t1_hh_rel=t1_hh_scst;<br>hh_hindu=max(t0_hh_hindu,t1_hh_hindu);<br>if hh_hindu=0 then hh_rel=2; else hh_rel=hh_scst;	religion
